# Moderating Effect of Interpersonal Mindfulness Between Marital Conflict and Marital Intimacy Among Iranian Married Individuals

**DOI:** 10.1002/brb3.70392

**Published:** 2025-02-28

**Authors:** Soolmaz Dehghanidowlatabadi, Harikumar Pallathadka, Sayed M. Ismail, Diyorjon Abdullaev, Fatma Magdi Ibrahim, KDV Prasad

**Affiliations:** ^1^ Department of Counseling, Faculty of Social Science Islamic Azad University, Science and Research Branch Tehran Iran; ^2^ Manipur International University Imphal India; ^3^ Department of English language and Literature, College of Science and Humanities Prince Sattam bin Abdulaziz University Alkharj Saudi Arabia; ^4^ Department of Scientific Affairs, Vice‐rector for Scientific Affairs Urganch State Pedagogical Institute Urganch Uzbekistan; ^5^ Department of Community Health Nursing RAK Medical and Health Sciences University Ras Al Khaimah UAE; ^6^ Geriatric nursing Mansoura University Mansoura Egypt; ^7^ Symbiosis Institute of Business Management (SIBM) Symbiosis International (Deemed University) (SIU) Hyderabad India

**Keywords:** interpersonal mindfulness, marital conflict, marital intimacy, married individuals, moderator

## Abstract

**Purpose:**

Marital intimacy is one of the significant factors determining life quality, which, along with interpersonal mindfulness, enables married individuals to experience greater levels of marital satisfaction. This study aimed to elucidate and confirm the moderating effects of interpersonal mindfulness in the relationship between marital conflict and marital intimacy.

**Method:**

The participants of this study comprised 207 Iranian married individuals (ages 19 to 45 years, mean age: 30, SD: ±4.54). Participants completed online questionnaires which assess interpersonal mindfulness, marital conflict and marital intimacy needs questionnaire.

**Finding:**

The results from structural equation modeling indicated that marital conflict negatively predicted marital intimacy (*β* = −0.484, *t* = 6.74, *p* <0.001), and interpersonal mindfulness positively predicted marital intimacy (*β* = 0.412, *t* = 5.14, *p* < 0.001). The findings from multigroup analysis demonstrated that interpersonal mindfulness moderated the relationship between marital conflict and marital intimacy in this study.

**Conclusions:**

The results of this study showed that interpersonal mindfulness as a moderator reduces the negative effects of marital conflict and increases marital intimacy among Iranian married individuals.

## Graphical abstract

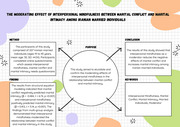

## 1 Introduction

So far, a large number of studies have shown that marriage contributes to improving people's health; however, it has been found that other factors besides marriage itself are involved in this matter (Carr and Springer 2010; Lee and Han 2023; Simon 2014; Tatangelo et al. 2017; Umberson and Karas Montez 2010). Marital intimacy (MI) is one of the important factors that may contribute to marital satisfaction and psychological well‐being (Aghamiri and Vaziri 2019; Nayeri et al. 2014). MI can satisfy people's psychological needs (Prager and Buhrmester 1998). To feel belonging, which is one of our needs (Glasser 1999; Maslow and Lewis 1987), we seek to establish intimate relationships. It can be said that one of our main incentives to get married is fulfilling intimacy (Petty 2010). MI is a multidimensional construct and expresses a deep level of emotional, physical, and psychological connectedness of the couple in marriage. MI involves the sharing of sexual experiences, the development of trust, and offering mutual support. MI manifests through open communications, emotional support, and frank sharing of personal thoughts and feelings (Adams and Jones 1997). Intimacy is a dynamic process and a product of closeness, a pleasant relationship, sharing thoughts, talking about private feelings and thoughts, and attention (Mohammadi et al. 2013). It is very important to consider intimacy in marriage because intimacy is one of the factors to prevent relationship dissolution (Minnotte et al. 2010; Mirgain and Cordova 2007). Since MI is an important factor in the strength of relationships, knowing the factors related to it can help to improve relationships. In this regard, we designed a study to examine the link between marital conflict (MC), interpersonal mindfulness (IM), and MI. Also for enriching our findings, the moderating role of IM was tested in the link between MC and MI.

Despite the amount of love between couples, MC is unavoidable. When two people get close to each other, some characteristics or personal habits of each may seem annoying for the other (Pathan 2015). Because MI provides a foundation for emotional closeness and mutual understanding between partners, it plays an important role in shaping relationship dynamics (Kardan‐Souraki et al. 2015). However, despite the presence of intimacy, conflicts could still arise due to differing expectations, stressors, and individual differences that challenge this closeness. Understanding how these conflicts emerge in the context of marital intimacy is essential because the flexibility and quality of intimate communication could significantly influence how couples navigate conflict (Habibi et al. 2024). This interplay between intimacy and conflict highlights the importance of interpersonal attention, which allows couples to constructively address differences while maintaining their emotional bond. Researchers have shown that by using methods to reduce marital conflicts, intimacy has increased (Davoodvandi et al. 2018; Yoo et al. 2014). Another research study showed that as marital conflicts increase, intimacy decreases significantly (Nikoogoftar 2021). But the question is, as the existence of conflicts in a relationship is natural, does the reduction of intimacy during conflicts also seem an obvious thing? The answer is that conflict resolution and intimacy styles are related (Prager 1991). John Gottman's marital stability theory and his ideas concerning the “Four Horsemen of the Apocalypse” (Gottman, 2019) and the therapeutic value of positive interactions have provided a strong basis through which MC and intimacy processes could be understood (Olson and Donahey 2017). According to, effective communication and emotional attunement are important in navigating conflicts in a manner that preserves MI. By incorporating interpersonal mindfulness (IM) into this framework, the ways in which couples could apply mindful awareness in effectively addressing those corrosive behaviors identified by Gottman—criticizing, being defensive, contempt, and stonewalling—can be elaborated. John Gottman (Navarra and Gottman 2013), in his theory, stated that masters of relationships (stable and happy couples) have the ability to remain positive about their partners, and they don't distort their partner's positive actions into neutral or even negative. According to this theory, as the relationship progresses, you map out what is going on in your partner's mind through asking him/her open‐ended questions. This is where IM becomes important. In the present study, we investigate the moderating role of IM in the relationship between MC and MI. As suggested by Gottman's theory (2019), the quality of interactions between partners directly influences relationship satisfaction; hence, IM could be a transformative practice that helps couples to transform potentially destructive conflicts into constructive conversations. This also aligns with Gottman's emphasis on the importance of positive interaction ratios and emotional support in maintaining a healthy marriage.

Recent studies have shown that mindfulness may also play an important role in interpersonal settings (Jones et al. 2019; Leavitt and Karremans 2023). Many relationship scientists hold that paying attention to and being aware of many of the processes that are essential to the health of intimate relationships (Adair et al. 2018). For instance, responding to your partner's demands is necessary to be attentive to them. Kabat‐Zinn (2015) defined mindfulness as paying non‐judgmental attention to current moment‐to‐moment experiences in situations and time, observing without passing judgment, and accepting one's thoughts and feelings. According to studies, mindfulness is positively correlated with interpersonal abilities, including compassionate communication, active listening, and building strong relationships with others (Abdollahi et al. 2022a). In this study, the interpersonal mindfulness scale developed by Pratscher et al. (2022) was used to measure interpersonal mindfulness, which includes four subscales: Presence (the capacity to attend to interpersonal interactions with complete awareness and accuracy), Awareness of self and others (the capacity to pay attention to one's own inner experiences and those of others, including non‐verbal behavior and intent), non‐judgmental acceptance (the capacity to accept and not prejudge interpersonal interactions), and non‐reactivity (the capacity to interact deftly and avoid snap judgments). IM could refer to the practice of bringing awareness and presence to interactions with others. In marriages, in respect of marital conflict and intimacy, partners have to be interpersonally mindful and heighten their awareness in conflicts to help them better receive the onslaught of their thoughts, emotions, and behaviors, thus responding to their partner in empathy and compassion instead of impulsively (Deniz et al. 2020). Hence, IM might ease the adverse consequences of conflict but allow the couples to learn deeper from the latter, strengthening the emotional connection. IM could be developed through practicing IM, since the more securely attached one is in their marital relationship, the more constructively he or she will deal with conflicts arising during the marriage. This may turn probably destructive conflicts into opportunities for personal growth and coming closer together, therefore strengthening the connection between partners (Pratscher et al. 2019). MC, where mindfulness is not used, fosters misunderstanding and emotional detachments thus compromising intimacy. With the awareness of interaction between the aforementioned components, resolution of conflict with IM may help in resolution not only at the present state but in its maintenance and perpetuation with the quality and depth of MI. Couples would also be able to deal with conflicts more constructively through the mindful approach to interpersonal interactions, maintaining the relationship resilient and intimate.

It is of particular importance to study MC, MI, and IM in Iranian culture because of its special cultural features. Traditionally, Iranian culture has been a collectivist culture with strong family values, which might affect marital expectations and behavior. Such cultural aspects may shape distinct ways in which couples manage conflicts and foster intimacy in their relationships (Kamali et al. 2020). Moreover, gender roles and social expectations in Iran could be influential in the quality of marital relationships and the nature of conflicts. Society may put pressure on couples to behave in a certain way in their interactions, and thus, these variables need to be analyzed within the Iranian cultural context. This can further our understanding of the challenges and opportunities that exist in Iranian marital relationships. IM as an empowering approach to handling conflicts and strengthening intimacy in marital relationships may provide effective ways of improving the quality of married life in this society (Nezhad and Goodarzi 2011). Thus, the interaction among these three variables could meaningfully contribute to strengthening marital relationships and enhancing family well‐being within the Iranian cultural framework.

### 1.1 Conceptual Framework

This study explores the moderating role of IM in the relationship between MC and MI in a sample of married individuals in Iran. The conceptual framework of this research relies on the fact that MC seriously deteriorates the quality of intimate relationships. MC is defined as every form of disagreement or tension between the partners, which may produce emotional hurt and a decline in relational satisfaction (Beyrami and Azhideh 2025). IM including non‐judgmental awareness during interaction moments‐may largely determine how individuals cope with conflict situations. IM may nurture non‐evaluative awareness of one's thoughts, emotions, and reactions in the middle of conflicts, which could enhance effective communication and empathy from a partner, and, thus, reduce the adverse effects of conflict (Pratscher et al. 2019). Therefore, the current conceptual framework considers IM as a moderator in the relationship between MC and MI. When levels of interpersonal mindfulness with partners are higher, lesser negative effects stemming from MC on MI could be seen. On the contrary, relatively low levels of IM may heighten the adverse effects between MC and MI. Therefore, this study hypothesized a significant relationship between MC and MI and hypothesized that IM moderates the relationship between MC and MI.

## 2 Methods

### 2.1 Participants

The participants in this study included 207 married individuals (women 137 and men 70) aged from 19 to 45 years (mean age: 30, SD: ±4.54). The age range included in the inclusion criteria was over the age of 18 years old. However, the reasons that could justify the age range of the study participants are as follows: This represents a broad range of marriage—from the newly married to the few who had been married for several years. The relatively young age of a number of the respondents allows this study to take into consideration any problems and processes that are generally associated with the early years of marital life, which may present very different issues relating to intimacy and conflict. Meanwhile, the responses from the more mature respondents would be adding in the insights pertaining to long‐term marital dynamics and stability, the changing relational needs. Furthermore, the investigation of a wide age range will increase the relevance and applicability of the study across various life stages, thus enabling wider understanding of marital conflict and intimacy within the Iranian cultural context. This inclusiveness will definitely contribute to the richness of the data collected and support the aim of identifying patterns and trends that may vary in relation to age and marital experience.

The average duration of marriage of the participants was 11 years (SD = ±4.5) with a range between 2 and 25 years. One hundred and twenty‐five of participants had a bachelor's degree, 35 of the participants had a diploma, 40 of the participants had a master's degree, and 7 of the participants had a PhD degree. One hundred and ten married individuals declared that they have no children, 62 of them declared to have one child, 20 of participants declared to have two children, and 15 of participants declared to have three children.

### 2.2 Procedure

The department of counseling at Azad Islamic University reviewed and accepted the study technique and ethical problems. The participant's consent form was included on the first page of the online survey, which was hosted on the Porsline platform. Participants were given access to the survey links via the social media platforms Telegram, WhatsApp, and ETA. The participants’ average response time to the questionnaires was 20 min, and the data‐gathering period ran from January 1 to February 30, 2023. The respondents were selected by using certain inclusion criteria: married, 18 years old and above, and living in Iran during the time of the study. Divorced, widowed, and those in a non‐marital status were excluded from the study, as well as those who did not give their consent to participate in this study.

### 2.3 Measures

**Interpersonal Mindfulness Scale** (IMS) (Pratscher et al. 2019) is a self‐report measure comprising 27 items across four subscales, including presence (7 items), awareness of self and others (10 items), non‐judgmental acceptance (4 items), and non‐reactivity (6 items). Items are scored on a 5‐point scale ranging from 1 (almost never) to 5 (almost always). The total score ranges from 27 to 135, with higher scores indicating greater levels of IM. Sample items include “*When I am with other people, I am aware of my moods and emotions*.” An Iranian version of the IMS was used in this study (Abdollahi et al. 2022b), and the Cronbach alpha value was 0.88.

**Marital Conflict Questionnaire‐Revised** (MCQ‐R; Sanai et al. 2000) is a self‐report measure consisting of 54 items across eight subscales, including the decrease in coordination (5 items), decrease in sexual intercourse (5 items), increase in emotional reactions (8 items), increase in seeking children's support (5 items), increase in personal relations with one's own family (6 items), decrease in family relations with the spouse's relatives and friends (6 items), separation of finances from each other (7 items), and decrease in effective relations (12 items). Items are scored on a 5‐point scale ranging from 1 (always) to 5 (never). The total marks range from 54 to 270, with higher scores suggesting greater levels of marital conflict. Sample items include “*While arguing with my spouse, my relation with his/ her family gets broken*.”

**Marital Intimacy Needs Questionnaire** (MINQ) (Bagarozzi 1997) is a self‐report measure containing 41 items across eight subscales, including emotional intimacy (items 1–5), psychological intimacy (items 6–10), intellectual intimacy (items 11–15), sexual intimacy (items 16–20), physical intimacy (items 21–25), spiritual intimacy (items 26–31), esthetic intimacy (items 32–36), and social‐recreational intimacy (items 37–41). Each item is scored on a 10‐point scale ranging from 1 (there is no such need) to 10 (there is a great need). The total score for each subscale except for the spiritual intimacy varies from 5 to 50, while the overall score of the spiritual intimacy ranges from 6 to 60. Higher scores in each subscale indicate greater levels of marital intimacy needs. Sample items include, “*How important is it to you that whenever you share your feelings with your spouse, they understand you and listen to you?*.”

### 2.4 Statistical Analysis

The software SmartPLS3 was employed because it can investigate proposed associations between variables with a small sample size, is non‐sensitive to normally distributed data, and can study the moderation model (Ringle et al. 2015). In this study, the measurement model was assessed by average variance extracted (AVE), composite reliability (CR), and Cronbach's alpha. In the structural model, the research hypotheses, coefficient of determination (*R* ^2^), effect size (*f* ^2^), and Stone–Geisser (*Q* ^2^) values were evaluated. For evaluating moderation analysis, multigroup analysis was employed (Henseler et al. 2015).

## 3 Results

### 3.1 Preliminary Analysis

Since participants completed the data online and all items were obligatory to be answered, no missing values were found in the preliminary analysis of the data. We further analyzed the dataset for outliers using Mahalanobis d square. The finding showed no values beyond the threshold of 4, thus indicating that extreme values did not influence the results. For normality, we used the measures of skewness and kurtosis. These showed that the data were normally distributed since the values for skewness and kurtosis were less than the cutoff scores ±2 and ±3, respectively (Hair et al. 2014).

### 3.2 Measurement Model

AVE was used to evaluate convergent validity, while CR and Cronbach's alpha were used to measure composite reliability (Henseler et al. 2015). Since the values of AVE, CR, and Cronbach's alpha were higher than the threshold scores of 0.5, 0.7, and 0.7, indicating sufficient convergent validity and reliability. Table 1 reveals appropriate convergent validity and reliability (Hair et al. 2014). Two metrics—the Heterotrait–Monotrait Ratio (HTMT) and the variance inflation factor (VIF)—were computed to assess discriminant validity and multicollinearity. The HTMT and VIF values were below the 0.85 and 5 criteria in Table 1, showing sufficient discriminant validity (Henseler et al. 2015).

**TABLE 1:** Values of composite reliability (CR), average variance extracted (AVE), heterotrait–monotrait ratio (HTMT), and variance inflation factor (VIF).

Variable	AVE	Cronbach's alpha	CR	HTMT	VIF
Interpersonal mindfulness	0/533	0/71	0/71	0/81	2
Marital intimacy need	0/659	0/73	0/73	0/50	0/10
Marital conflict	0/543	0/72	0/72		1

### 3.3 Structural Model

The hypotheses, coefficient of determination (*R* ^2^), effect size (*f* ^2^), and Stone–Geisser (*Q* ^2^) values were evaluated at the structural model stage (Henseler et al. 2015). The results of the structural model showed that MC negatively predicted MI (*β* = −0.484, *t* = 6.74, *p* < 0.001) and IM positively predicted MI (*β* = 0.412, *t* = 5.14, *p* < 0.001). To assess the variance in MI by MC and IM, the coefficient of determination (*R* ^2^) was used. *R* ^2^ for MI was 0.364, indicating MC and IM explained 36.4% of the changes in marital intimacy. This value was higher than the cutoff of 0.35, which is considered a moderate coefficient of determination (Henseler et al. 2015). The effect size (*f* ^2^) was used to quantify each predictor's contribution to the outcome variable. Large effect sizes (> 0.35) are needed to explain marital intimacy, as shown by the effect sizes (*f* ^2^) for MC and IM, which were 0.38 and 0.39, respectively (Cohen 1988). The Stone–Geisser (*Q*_2_) value was 0.38, which is higher than 0.35 and indicates that MI has a high predictive relevance (Henseler et al. 2015).

### 3.4 Moderating Role of IM

To test the moderating role of IM, we used Partial Least Squares Structural Equation Modeling, PLS‐SEM, as the statistical technique. The multigroup analytic approach was used to determine whether IM had a moderating effect on the link between MC and marital intimacy. Initially, individuals were divided into two groups based on IM cut‐off scores: low IM group (30–74) and high IM group (74.1–125). We used the median as the cutoff point to define the low IM and high IM groups. More precisely, participants who scored at or below the median of 74 were classified as the low IM group, while those who scored above the median were classified into the high IM group. The rationale for using the median is providing a clear‐cut division between the lower and upper halves, thereby classifying the participants in a balanced way according to their level of IM. The underlying logic of the multigroup analysis for testing moderation is that if a relationship between the predicting variable (MC) and the outcome variable (MI) is significant in one group and not significant in another group, then it can be concluded that the moderator (IM) has played a moderating role. That suggests that the moderator has weakened or strengthened the association between the two variables. Multigroup analysis results for these groups with varying IM levels (low group: *β* = −0.487, *t* = 4.893, *p* < 0.001 and high group: *β* = −0.107, *t* = 1.87, *p* > 0.05) indicated that the relationship between MC and MI weakens as IM increases. Therefore, IM served as a moderator in the association between MC and marital intimacy.

## 4 Discussion

The first hypothesis of this survey was to test the relationships between IM, MC, and MI among Iranian married individuals. MC negatively predicted MI. A possible explanation for the negative relationship between MC and MI could be that MC may cause anger, hostility, revenge, hatred, jealousy or physical and verbal abuse between couples (Molajafar et al. 2015). When couples have conflicts, emotional regulation in themselves and their partners is disturbed, and they may show impulsive reactions and less positive emotions, and they are more likely to suffer from interpersonal problems (Skoranski et al. 2019). During conflicts, couples often suffer a loss in their ability to effectively manage both their partner's feelings. According to a study conducted by Skoranski et al. (2019), such emotional dysregulation during times of difficulty can cause impulsive actions, suppression of positive feelings, and a heightened chance of encountering interpersonal complications. In cultures such as Iran, in which expression of emotion can be closely monitored and regulated, individuals can become unable to verbalize feelings during times of conflict and therefore contribute to increased miscommunication and heightened tension (Kakolian et al. 2024). Moreover, in cultures that maintain traditional gender roles, expression of conflict can become gender‐dependent. For example, men can become forced to exhibit dominance in times of conflict and therefore become abusive, and women can opt not to speak and withdraw, both of which can hinder feelings of closeness and affiliation. All these actions together form a destructive feedback loop of actions, eroding the basis of intimate marriage. The intersection of these behavior and emotion dynamics culminates in a loss of MI, with couples trapped in a loop of destructive behavior that destroys feelings of closeness and fondness. Suffering produced through unresolved conflicts can cause a retreat from intimacy, and couples will then become conditioned to view contact with hurt, not comfort (Behrang et al. 2022). These negative emotional characteristics and harassment between couples may lead to less MI between couples.

The second aim of this study was to test the moderating role of IM in the link between MC and MI. The findings from multi‐group moderation analysis showed that MC is more likely to less MI when there is an absence of IM. IM is associated with characteristics such as observing, describing, and not judging in relationships with others. These characteristics of IM may contribute to having greater levels of empathy and understanding other's emotions, and individuals could regulate their own and other's emotions, and use efficient behaviors instead of conflicting with others (Jones et al. 2019). Individuals with high IM are more likely to accept the negative characteristics of their couples and have less conflict with couples (Jones et al. 2019). People with greater IM also have high compassion towards others, and this compassion helps them to be aware and regulate others’ emotions and helps them to have more empathy with others instead of conflict (Skoranski et al. 2019). Thus, IM shows promise in helping couples manage their conflict responses. Therefore, when couples speak with each other in an atmosphere of openness and not criticism, such a dialogue may promote heightened intimacy and ease in marriage tension. As such, IM could be seen as a valuable concept that helps couples manage their conflict reaction in a manner that promotes a supportive environment for development and empathetic listening. Moreover, by lessening cognitive loads and allowing for reflection, IM enables couples' access to the current moment, and therefore increased relationship satisfaction and intimacy (Pratscher et al. 2019).

The results of this study highlight the roles of IM and MC in predicting MI in married individuals. Therefore, the evaluation of IM and MC in married individuals are significant activities for psychologists and counselors because MI plays a key role in the couples’ marital satisfaction.

Although these results are positive, it is important to recognize some restrictions. Self‐report questionnaires were used in this study, and such a basis could introduce social desirability and recall bias. Therefore, causal inference regarding relationships between variables was not permitted. Biases may impact the accuracy of the measured levels of MC, MI, and IM, and in such a manner, restrict the generalizability of the findings. The data of this study was collected via an online survey, and it is possible that the studied sample is not a true representative of the studied population. It is possible that the respondents might show differences from the larger population in important characteristics, such as demographics or relationship status, thus potentially limiting the generalizability of the results.

To overcome the limitations found in future studies, it is recommended to use a wide range of data collection methods, such as observational studies or dyadic interviews, which could potentially provide a deeper and richer analysis of the interactions between couples. The use of objective measures, such as assessment scales filled out by independent raters, could also enhance the generalizability of the findings. Longitudinal studies would be beneficial in determining causality and tracking changes over time, thus allowing for a better understanding of the impact of IM on MC and MI. Overall, a mixed‐methods approach could offer a deeper and more holistic understanding of the intricacies involved in marital relationships.

In conclusion, our findings imply that IM may increase MI, whereas MC may contribute to less marital intimacy. Our results also emphasize the moderating impact of IM for understanding when the association between MC and MI is strengthened or weakened. It is important to consider the practical implications for couples and treating clinicians. Developing mindfulness skills could act as a useful prevention or intervention for couples wishing to develop a healthy relational life, particularly in terms of resolving conflicts and enhancing intimacy.

## Author Contributions

**Soolmaz Dehghanidowlatabadi**: conceptualization, methodology, data curation, formal analysis, writing–original draft. **Harikumar Pallathadka**: data curation, methodology, writing–original draft. **Sayed M. Ismail**: conceptualization, formal analysis, data curation, methodology. **Diyorjon Abdullaev**: writing–original draft. **Fatma Magdi Ibrahim**: writing–original draft. **K. D. V. Prasad**: writing–review and editing.

## Ethics Statement

The study procedure was approved by the ethics committee of the Azad Islamic University (IR/11/27/1401) and informed consent was obtained from the participants. All methods have been carried out in accordance with relevant guidelines and regulations.

## Consent

Informed consent was obtained online from all participants.

## Conflicts of Interest

The authors declare no conflicts of interest.

### Peer Review

The peer review history for this article is available at https://publons.com/publon/10.1002/brb3.70392.

## Data Availability

The data that support the findings of this study are openly available in Figshare at https://figshare.com/account/items/23690577/edit, reference number 10.6084/m9.figshare.23690577.
